# Unveiling intimacy: sexual dysfunction and marital satisfaction among Pakistani males in Karachi

**DOI:** 10.1093/sexmed/qfae070

**Published:** 2025-01-13

**Authors:** Hayat Ali Yousefzai, Siti Irma Fadhilah Ismail, Sana Hussain, Aishah Siddiqah Alimuddin

**Affiliations:** Department of Psychiatry, Faculty of Medicine and Health Sciences, Universiti Putra, Malaysia; Department of Psychiatry, Dr. Ziauddin University Hospital Karachi, Pakistan; Department of Psychiatry, Faculty of Medicine and Health Sciences, Universiti Putra, Malaysia; Department of Psychology, Faculty of Social Sciences Shaheed Zulifiqar Ali Bhutto Institute of Science and Technology, Karachi, Pakistan; Department of Psychiatry, Faculty of Medicine and Health Sciences, Universiti Putra, Malaysia

**Keywords:** sexual dysfunction, marital satisfaction, male adult Pakistan, psychosocial dynamics, openness, knowledge gaps, health services, individual consequences, sexual health, thematic analysis

## Abstract

**Background:**

In Asian countries, discussing sex-related issues remains a taboo. Sexual dysfunction is not even considered a serious disorder in Pakistan.

**Aim:**

To explore sexual dysfunction and marital satisfaction within the Pakistani context to develop supportive intervention programs,

**Methods:**

This study entailed a mixed method approach and was carried out in Karachi, Pakistan. The sample comprised 28 married men, and data were gathered by in-depth interviews. Subsequently, data were analyzed through content analysis.

**Outcomes:**

There is a strong relationship between marital dissatisfaction and sexual dysfunction in adult married males aged 25 to 40 years in Pakistan.

**Results:**

The analysis of participants’ perspectives revealed 5 themes: psychosocial issues, rationale of openness in marital life, insufficient sexual knowledge, lack of sexual health service, and individual consequences.

**Clinical Implications:**

The results of this study suggest that it is an important suggestion to the government of Pakistan to introduce sexual health counseling and premarital counseling programs at the university level. The Ministry of Health and Higher Education needs to promote awareness about sexual health, sexual dysfunctions, and marital satisfaction, which will enable men to understand their sexual problems and effectively cope with them.

**Strengths and Limitations:**

The results of this study highlight the biopsychosocial dimensions of human sexuality. In this context, the psychosocial aspects associated with sexual dysfunctions are influenced by cultural and societal norms, where open discussions about sexual issues between male and female partners may be limited due to concerns about maintaining harmony in marital relationships. The limitation of this study is that the sample is not generalized; it is also not a demographic representation of all socioeconomic groups in Pakistan. Participants in low and middle classes reported an inability to seek help from professionals due to the high costs of treatments. Therefore, the results cannot to be extended to all Pakistani males.

**Conclusion:**

In this study, male sexual dysfunction strongly affects marital satisfaction within the couple. As such, marital counseling and psychotherapeutic strategies play an important role to help individuals and couples manage their sexual dysfunctions and enhance their marital satisfaction.

## Introduction

Marriage satisfies a basic human need for emotional support and companionship.^1^ Studies reveal that stable marital relationships considerably enhance psychological health by offering a sense of security and belonging.^2^ Marital satisfaction can be defined as the degree to which spouses feel happy and pleased in their partnership.^3^ It can be affected by numerous factors, including the quality of the relationship, communication patterns, unique attributes of partners/spouses, and effective problem-solving strategies.^4^ Moreover, sexuality and sexual issues have a profound impact on the dynamics of relationships within couples.^5^ For men’s overall psychological health, sexuality is crucial. Most men experience numerous sexual diseases, and many have sexual dysfunction.^6^ Sexual dysfunction encompasses a range of illnesses, including poor, premature ejaculation delayed ejaculation, sexual desire, and erectile dysfunction. The inability to completely participate in gratifying sexual involvement is a typical description of sexual dysfunction.^7^ It refers to conditions that make it challenging to engage in and enjoy sexual activity and that interfere with the regular sexual response. Although these conditions rarely pose a threat to physical health, they can have detrimental psychological consequences, such despair, anxiety, helplessness, and decreased productivity.^8^ Marital dissatisfaction and psychological distress are unavoidable problems in a married relationship. Marital dissatisfaction frequently causes divorce and separation. In this regard, it is essential to investigate practical coping strategies to handle these issues.^9^

In Asian countries with Islamic populations, discussing sex-related issues remains a taboo. Sexual dysfunction among Asian men is frequently overlooked and untreated due to conservative cultural and religious values, socioeconomic circumstances, and insufficient awareness.^10^ Even sexual dysfunction is not considered a serious disorder in Asian societies; instead, it is often viewed as a natural part of the aging process. As a result, this issue remains unaddressed. Few research studies have been carried out on sexual dysfunction within the Pakistani context.^11^ However, a multicenter study revealed that the prevalence of erectile dysfunction was highest (80.8%) among Pakistani men as compared with those in Egypt (63.6%) and Nigeria (57.4%).^12^ Given this, it is imperative to explore this phenomenon within the Pakistani context to develop supportive intervention programs, such as education, sex therapy, and problem-solving strategies, to improve the overall male’s sexual health and reduce marital dissatisfaction. The general objective of the study is to explore the relationship between sexual dysfunction and marital satisfaction in Pakistani men, whereas the specific objectives are to determine the prevalence of sexual dysfunction, assess the level of marital satisfaction, and identify demographic and psychosocial factors that influence marital satisfaction and sexual dysfunction in Pakistani married males.

## Methods

### Setting

The research was conducted in Karachi, Pakistan, which is the largest city in the country and ranks as the third largest city globally. Urdu serves as the official language of Pakistan, commonly spoken by the residents of Karachi. The study took place at Ziauddin University Hospital in Karachi, known as the largest hospital in Pakistan, housing 5 campuses across the city. Among these campuses, only the Ziauddin KDLB campus offers premarital counseling services within the psychiatry department. Urban and rural engaged couples, particularly males, often seek premarital counseling services from psychologists at this campus.

### Design

The research was conducted over 3 months, from June to August 2023, at the counseling clinic at the KDLB campus.

This qualitative study was conducted as part of a comprehensive exploration, and the objective of this qualitative study is to explore the experiences and perceptions of Pakistani married men regarding the relationship between sexual dysfunction and marital satisfaction. The decision to employ a qualitative design for this segment of the study was driven by the sensitive nature of the topic. Qualitative methods are effective for understanding complex human issues and their underlying factors, especially when there is limited prior knowledge and a need for thorough exploration.^13^

### Sample and inclusion/exclusion criteria

The study involved 28 engaged men chosen through purposeful sampling, ensuring diversity in factors such as age, education, socioeconomic status, and residency (urban or rural). Participants volunteered for counseling at the hospital and met specific inclusion criteria: fluency in Urdu, engagement status, absence of chronic diseases or mental illnesses, and willingness to participate. Participants were also inquired about being sexually active and their frequency of intercourse. Moreover, due consideration was given to participants’ religiosity and frequency of practice.

Data collection utilized semistructured face-to-face interviews conducted in appropriate settings. Participants completed a demographic questionnaire before interviews, covering age, gender, education, occupation, and residency. Interviews began with general questions, followed by inquiries about the impact of sexual dysfunction on marital relationships. Participants shared their experiences and views, with subsequent questions tailored to their responses.

The interviews were recorded with a digital voice recorder. Each interview lasted between 35 and 45 minutes. Data collection persisted until reaching data saturation, meaning that additional data did not introduce new themes or alter the existing ones.^14^ Following each interview, a verbatim transcription was promptly generated. For immersion in the data, the interview transcription was reviewed several times, hand coded by 2 independent reviewers, and compared for consistency of repeated observations.

### Data analysis

The interviews underwent analysis via the qualitative content analysis approach. Given its prominence among researchers for identifying themes, content analysis was chosen as the appropriate method for analyzing the study data.^15^ The researcher transcribed the recorded interviews entirely, following Miles and Huberman’s coding suggestions.^16^ Common concepts were coded to generate themes, categorized into major themes and subthemes. These themes and subthemes were compared and analyzed to determine their significance, leading to refinement and interpretation for content meaning. To identify the meaning unit, each interview was carefully reviewed, encompassing all significant points related to the importance of sexual dysfunctions and marital dissatisfaction. These segments were considered the meaning unit. Subsequently, the meaning units were thoroughly reviewed and coded according to their conceptual content. Similar codes were then combined and grouped by their similarities. The researcher analyzed the transcripts to identify primary response patterns, as well as consistencies and disparities among participants.

### Ethical considerations

Ethical considerations, including conflict of interest, misconduct, coauthorship, and double submission, were meticulously addressed. The research team prioritized participant rights by providing a clear explanation of the study’s purpose, ensuring privacy and confidentiality of information, and allowing participants to withdraw from the study at any time. Written informed consent was obtained from all participants prior to their involvement in the study. Moreover, the Ethics Committee of Universiti Putra Malaysia approved the subject study within or out of Malaysia and as an independent consultant at Ziauddin University Hospital Karachi; all participants gave consent to voluntarily join the study for research purposes. Their confidentiality was maintained, and the consultant provided a precounseling session after the administration of survey/questionnaire.

The sociodemographic variables of this study are as follows:

*Age:* Participants aged 25 to 40 years were considered for the study.

*Education:* Participants were inquired about their levels of education (ie, primary, middle, intermediate, or higher studies).

*Employment status:* Participants were inquired about their employment status (ie, unemployed, government employee, or self-employed).

*Residency:* Participants were inquired about their residency (ie, urban or rural).

## Results

The ages of participants are between 25 and 40 years. The characteristics of participants are presented in Table 1. Table 2 shows the perceptions of participants regarding sexual dysfunctions and marital dissatisfaction. These experiences are categorized into 5 themes and 16 subthemes.

**Table 1:** Sociodemographic variables for the lay informants (N = 28).

**Variable**	**No. (%)**
Age, y, mean (range)	31.7 (25–40)
Education	
Primary	3 (10.7)
Middle school education	4 (14.3)
Intermediate education	10 (35.7)
Higher education	11 (39.3)
Employment status	
Unemployed	13 (46.4)
Government employee	9 (32.1)
Self-employed	6 (21.4)
Residency	
Urban (town)	19 (67.9)
Rural	9 (32.1)

**Table 2:** Themes and subthemes in this study.

**Themes**	**Subthemes**
Psychosocial issues	Sociocultural aspects, family problems (eg, lack of social support), substance abuse due to cultural resistance, divorce, sexual risk-taking behavior (child abuse, even cases of rape)
Rationale of openness in marital life	Lack of communication, understandings of sexual feeling
Insufficient sexual knowledge	Insufficient formal education, absence of parental education, and limited knowledge of educators
Lack of sexual health services	Lack of expert workforces, high cost of consultation, and lack of priority in policy making
Individual consequences	Suicide attempts, marital dissatisfaction, low sexual confidence

### Psychosocial issues

Twenty-four participants (middle to higher education, government employees, and self-employees belonging to urban and rural areas) believe that Pakistan has a gap between the traditional and modern lifestyle in terms of marital life. The current study revealed a notable conflict between traditional and modern values, resulting in growing psychosocial issues. One participant said, “Some partners are confuse about sharing sexual dysfunctions with wife is not a masculine and she will think negative about him and this will lead psychological and sociological dysfunctions in society” (38-year-old).

In this regard, a participant noted, “They are already facing difficult condition and also experience social degrade among peers; so, if they discuss with wife may she also degrade him and will never support him in society due to this fear he feels un-compatible in family relationship with wife which leads him towards psychological distress” (a 25-year-old). Twenty-six participants shared similar thought patterns in this regard.

Fifteen participants believed that a lack of strong religious beliefs can have negative effects on sexual health, as they used masturbation in young age and extramarital affairs led them toward sexual dysfunctions.

### Rationale of openness in marital life

Eighteen participants (middle to higher education, government employees, and self-employees belonging to urban areas) believed that a large percentage of young people are currently facing problems related to openness in communication, a lack of communication, and understandings of sexual feelings between the partners, which minimized their communication at risk level. It make the young man engaged in extra usage of Internet and lead to cell phone abuse. This abuse was described by a participant as follows: “searching websites since they are more attractive, chatting, and online dating are also very popular in society which decreased the communication among couple” (25-year-old).

Twenty-five participants (primary to higher education, government employee, unemployed, and self-employed belonging to urban and rural areas) mentioned the harms caused by cell phone abuse: “They send private photos via applications such as Viber, messengers, and WhatsApp,” Sometimes the male partner gets blackmailed and due to guilt they never share this issue with wife. Male partners also have this fear that if they share this issue then wife will demand for divorce. Therefore, it leads to gap of communication among partners (33-year-old).

One of the points repeatedly expressed by many participants that was the harm caused by satellite and television channels in Pakistan. In this regard, 3 participants shared view that “The effect of satellite and TV channels cannot be ignored; it’s also play and show erotic and un-respectful series and dramas which also put wives in doubt and she will never discuss due to gap of communication” (38-year-old, 35-year-old, 32-year-old).

### Insufficient sexual knowledge

Seventeen participants (primary to intermediate education, government employee, unemployed, and self-employed belonging to urban and rural areas) stated that most young adult males did not have enough knowledge about their sexual health nor did they emphasize improving their sexual knowledge for the marital life cycle. Therefore, a lack of formal education is one of the main reasons for their insufficient knowledge in Pakistani society. In this regard, one of the participants said, “Officially, we still do not see anything in our educational system about these issues the sexual health counseling should add as subject in degree level formal education at universities in Pakistan” (a 39-year-old medical doctor).

Other participants mentioned that insufficient sexual knowledge was attributed to the existence of unreliable internet sources (eg, Bengali Baba’s, Mulvey’s, Tweezes). One participant disclosed, “I talked to one of my married friends regarding the sexual health problems in our society he said that reality is something else” (28-year-old school teacher).

Another participant noted, “Most of Pakistan’s families are having strict environment and feeling ashamed of talking about sexual issues with their children; if children ask something about sexual issues the parents responded they have not heard anything about this, because the parents believe that when children aware about sexual issues, it would be hard to control them and decreased the gap between parenthood” (33-year-old government employee).

### Lack of sexual health services

In this study, all participants were concerned with the lack of significance for sexual health in health policy making. In this regard, a participant stated, “Many of the problems with which we are facing now are related to lack of support from health policy maker” (31-year-old private employed).

Two participants said, “That considering these things as anti-Islamic agenda and spread obscenity so many people avoid talking about their sexual problems and they live with their problems for many years which cause marital dissatisfaction among the male in Pakistan” (35-year-old private teacher, 38-year-old clerk).

Most participants reported that the high cost of consultation services is preventing them from getting proper psychosexual health consulting services, which leads to martial dissatisfaction due to sexual dysfunction. “So, the cost of consultation is too high and the consultants are mostly setting in private setup they charge the clients too high fees” (32-year-old self-employed).

A few participants emphasized the lack of expert workforces in the sexual health field: “We haven’t come across any recognized experts in the field of sexual health in Karachi. If there are any, they often lack expertise and end up wasting people’s time due to their unfamiliarity with the fundamental terminology of sexual health in Pakistan” (33-year-old self-employed).

### Individual consequences

Sexual dysfunction among men in Pakistan, who have limited access to sexual and psychological health counseling and few economic resources, causes severe negative consequences in terms of depression, anxiety, and stress, leading to suicide attempts. One of the participants said, “At the time of intercourse if the male did not perform well in front of wife and didn’t get sexual pleasure at that time male partner feels empty, feels severe depress, feeling loneness and didn’t want to live anymore wanted to end his life; however, some of people in young age may committed suicides in such a condition” (26-year-old).

Moreover, participants expressed their primary concern regarding the prevalence of sexual dysfunction in men, which consequently contributes to marital dissatisfaction within relationships. One participant stated, “If the female partners are not fully sexually satisfied so like in these cases marital dissatisfaction may be entailed to male which social pressure and poor psychological wellbeing lead him towards suicides” (30-year-old).

The participants believed that many young men now lack sexual confidence in Karachi due to the unavailability of healthy food and their excessive usage of the internet for pornographic material. Furthermore, given that they have limited knowledge about sexual relationships and sexual health, their sexual confidence has also decreased. One participant noted, “Some of young male compare themselves with actors in pornographic movies and, then, they think they have sexual problems and lose their confidence in the time sexual relationship” (35-year-old business man).

## Discussion

This is the first qualitative study in Karachi, Pakistan, that explains the importance of providing awareness about sexual dysfunctions and marital dissatisfaction in married males. Data analysis resulted in 5 themes: psychosocial issues, rationale of openness in marital life, insufficient sexual knowledge, lack of sexual health service, and individual consequences.

Psychological issues, as explained by most participants, are the main reasons for marital dissatisfaction in men, so it is important to provide sexual health counseling related to sexual dysfunction to engaged couples. Most participants explained that Pakistanis are currently facing significant challenges in choosing between culture and a modern lifestyle, which could be the reason for the psychosocial issues, which create a threat for the sexual health between the couple in Pakistani society. Propagation of modern culture and ideas has changed Pakistani sociocultural values and caused a conflict between Pakistani culture and a modern lifestyle, which is formed in different approaches to psychosocial issues, such as sexual dysfunction, marital dissatisfaction, cultural resistance, and a lack of family support in males, potentially leading to divorce and abusive sexual behavior.^17^ The majority of participants noted that weak religious and cultural beliefs pose a potential risk to male sexual health, a concept explored by Michael et al, who reported that a lack of religious beliefs can contribute to deviations in sexual behavior.^18^ Additionally, many participants highlighted the increased prevalence of high-risk sexual behaviors as a threatening factor for sexual dysfunction.^19^ This escalation in risky behaviors could exacerbate communication barriers and the lack of openness in couples’ sexual lives, leading to adverse consequences. In this study, our findings align closely with those suggesting that extensive use of the internet, social media, cell phones, and television has significantly exposed young individuals to risky sexual behaviors.^20^

Most countries in the world have started to ban certain apps and websites spreading obscenity and harming the minds of people. However, the government of Pakistan has taken no specific long-term planning and prevention measures to stop the misuse of technology, which resultantly affects the sexual and marital lives of individuals. Per Takao et al,^21^ if technologies are misused, individuals may experience adverse effects on their sexual lives. Thus, implementing a sexual counseling educational program could prove highly effective for young men in averting the detrimental impacts of technology on sexual health and marital satisfaction. Yet, modern technology can be an opportunity as well as a threat; so, strategies need to be formed to use technology in a way that improves sexual health and well-being.

In this study, participants themselves identified insufficient sexual knowledge as a factor contributing to sexual dysfunction. This aligns with the findings of other researchers who have highlighted the lack of sexual knowledge as a significant factor in sexual dysfunction.^22^ Various reasons contribute to this lack of sexual knowledge, including inadequate formal education, reliance on unreliable sources, absence of parental guidance, and limited expertise among educators. Our study findings revealed that inadequate sexual knowledge—coupled with attitudes toward sex, misinformation, and reliance on unreliable sources such as YouTube and certain social media platforms—contributes to misconceptions about sexual issues among young people. This lack of awareness about sexual matters can lead to marital dissatisfaction in their future lives. Unfortunately, in Pakistani culture, parents typically feel uneasy discussing sexual dysfunction and related matters with their children. Consequently, parents often prove to be ineffective sources of information for addressing their child’s sexual issues and concerns, leaving the child to seek information from unreliable sources.^23^ Most of the time, such unreliable sexual knowledge sources cause misconceptions about sex, and that incorrect information in children’s minds leads to marital dissatisfaction later in life.^24^ So, many taboos, cultural beliefs, and traditions may stop young men from accessing the right information about sexual health.^25^ Although sexual health counseling education and premarital counseling are important in Pakistani society, there is still no comprehensive and formal education for engaged couples in Pakistan. Therefore, the government of Pakistan needs to pay attention to such an issue to prevent young men in Pakistan from marital dissatisfaction.

Most participants consider that a lack of sexual health services is a threatening factor for sexual dysfunction in Pakistan. Our study findings closely mirror those indicating a substantial disparity in Iran regarding the provision of sexual health services, including intervention strategies aimed at enhancing sexual health.^26^

Likewise, Pakistani citizens, particularly young men, lack sufficient sexual knowledge, information, and access to adequate sexual health services within Pakistan. The government’s failure to prioritize individual sexual health is among the factors hindering the provision of improved sexual health services to the population. Sexual health educational programs should be supported like other educational programs at the university level at the least.^27^ Therefore, government officials and health policy makers need to formulate long-term plans for introducing sexual health programs. Sexual health counseling programs should be made mandatory before marriage to prevent marital dissatisfaction, divorce, and suicide in Pakistani society.

In this study, the participants discussed some of the individual consequences of sexual dysfunction, which leads men toward divorce, suicide attempts, marital dissatisfaction, and low sexual confidence. A study concluded that divorce is also caused by sexual dysfunction in Pakistan,^28^ given that increased sexual satisfaction correlates with increased marital satisfaction and overall life happiness, coupled with the observation that sexual dissatisfaction contributes to >50% of divorces.^29^ Therefore, based on the current study, culturally sensitive sexual health education programs and premarital counselling should be considered as part of university curricula in Pakistan.

## Conclusion

The present study shows a strong relationship between sexual dysfunction and marital dissatisfaction in married men in Pakistan. Psychological, sociological, cultural, and health-related factors have an undeniable impact on sexual dysfunction leading to marital dissatisfaction. Moreover, a lack of communication and understanding in married couples, misconceptions about sexual health, excessive use of the internet as well as social media and obscene content, and religious aspects aggravate sexual dysfunction in Pakistani married males. Our findings suggest that the government of Pakistan should introduce sexual health counseling and premarital educational programs at the university level. The Ministry of Health and Higher Education should prioritize raising awareness about sexual health, sexual dysfunctions, and marital satisfaction. This effort will empower men to comprehend and manage their sexual issues effectively. This sexual health awareness and counseling at the university level will prevent a higher rate of suicide due to marital dissatisfaction in young married males. Moreover, further investigation in this domain has the potential to enhance marital satisfaction and reduce divorce rates among the Pakistani populace. It is pertinent to mention that the study encompasses only a small sample of the population due to the fact that the subject matter is still considered a taboo and sensitive topic of discussion in Pakistani society; therefore, not many people are willing to discuss and share it openly or even with health specialists. Thus, there is a dire need for more discussion and research on this topic, which should include women and variables such as religion.
